# mHealth Intervention for Elevated Blood Pressure Among College Students: Single-Arm Intervention Study

**DOI:** 10.2196/48520

**Published:** 2024-06-07

**Authors:** Dieu-My Tran, Catherine Dingley, Roger Bonilla

**Affiliations:** 1 School of Nursing University of Nevada, Las Vegas Las Vegas, NV United States

**Keywords:** blood pressure, mHealth, self-management, students, intervention, elevated blood pressure, college, hypertension, young adult, mobile app, smartphone, monitoring, text messaging, text mining

## Abstract

**Background:**

Current evidence reveals a growing pattern of hypertension among young adults, significantly increasing their risk for cardiovascular disease later in life. Young adults, particularly those of college age, often develop risk factors related to lifestyle choices in diet, exercise, and alcohol consumption. Developing useful interventions that can assist with screening and possible behavioral modifications that are suitable and appealing to college-aged young adults could help with early identification and intervention for hypertension. Recent studies indicate mobile health (mHealth) apps are acceptable and effective for communication and message delivery among this population.

**Objective:**

The purpose of this study was to examine the feasibility of using a mobile smartphone delivery system that provides tailored messages based on participant self-measured blood pressure (BP) with college-aged young adults.

**Methods:**

Using a single-arm intervention, pilot study design, the mHealth to Optimize BP Improvement (MOBILE) intervention was implemented with college students aged 18 years to 39 years who had systolic BP >120 mm Hg and diastolic BP ≥80 mm Hg. Participants were required to measure their BP daily for 28 days, submit the readings to the app, and receive preset educational text messages tailored to their BP value and related to encouraging healthy lifestyle modifications. Changes in a participant’s BP was evaluated using a mixed regression model, and a postintervention survey evaluated their perspectives on the mHealth intervention.

**Results:**

The participants’ (N=9) mean age was 22.64 (SD 4.54) years; 56% (5/9) were overweight, and 11% (1/9) were obese. The average daily participation rate was 86%. Of the 9 participants, 8 completed the survey, and all indicated the intervention was easy to use, found it increased awareness of their individual BP levels, indicated the text messages were helpful, and reported making lifestyle changes based on the study intervention. They also provided suggestions for future implementation of the intervention and program. Overall, no significant changes were noted in BP over the 28 days.

**Conclusions:**

The mHealth-supported MOBILE intervention for BP monitoring and tailored text messaging was feasible to implement, as our study indicated high rates of participation and acceptability. These encouraging findings support further development and testing in a larger sample over a longer time frame and hold the potential for early identification and intervention among college-aged adults, filling a gap in current research.

## Introduction

### Cardiovascular Risk Factors

Cardiovascular disease (CVD) remains one of the leading causes of death in the United States, with 840,768 deaths reported in 2016 [1]. Based on the projections for 2019 onward, cardiac events are predicted to occur in 1,055,000 individuals, 720,000 of which will be new cases [2]. Additionally, from 2014 to 2015, the reported annual total cost for CVD-related treatments was 351.2 billion, and this is projected to increase to $1 trillion by 2035 if current trends persist [3]. Chief among CVD risk factors is high blood pressure (BP), with 63% of CVD-related events occurring in individuals with a systolic BP (SBP) ≥140 mm Hg and diastolic BP (DBP) ≥90 mm Hg [4]. The prevalence of high BP (SBP ≥140 mm Hg) has increased steadily since 1990, with an estimated 3.47 billion adults with hypertension worldwide as of 2015 [5].

### Background

The majority of studies focused on hypertension have involved older adults; however, recent research reveals a growing pattern of hypertension among young adults, starting as early as childhood [6]. The prevalence of hypertension was estimated to be 29.0% in 2015-2016, with increasing prevalence by age; specifically, in young adults aged 18 years to 39 years, the prevalence was 7.5%. Men (9.2%) have a higher prevalence of hypertension than women (5.6%) during young adulthood (18-39 years); however, as they become older adults, women catch up, with a higher prevalence of hypertension than men [7]. Unfortunately, the prevalence of hypertension has increased in the young adult population. According to National Health and Nutrition Examination Survey 2015-2016 data, 13.3% of children and adolescents 8 years to 17 years old had elevated BP, while the 2017-2020 data demonstrated that young adults between the ages of 20 years and 44 years had a 28.5% prevalence of hypertension [6]. Based on the 30-year Framingham risk score, 17.3% of men and 9.2% of women in the National Longitudinal Study for Adolescent Health (n=14,333) were at risk of developing general CVD complications later in life [8]. Despite these findings, among adults 20 years to 45 years old, less than 50% of women and less than 40% of men are screened for risk factors [9]. Early screening becomes increasingly critical, as early interventions, such as dietary and behavioral changes, in early adulthood can prevent as much as 80% of cases later in life [10]. Screening is acknowledged for its importance; however, due to the increasing prevalence of hypertension in this population, intervention is warranted to delay or prevent the increase in hypertension development during young adulthood.

Targeting young adults of college age is particularly significant, as many of the risk factors for high BP commonly start during this period of life. Studies have shown the transition to college life is often associated with poor dietary choices such as reduction in fruit and vegetable consumption; increased consumption of sugary drinks; and high-fat, high-sodium food consumption [11]. On average, college students gain 6 pounds over the course of a 4-year education [12]. Additionally, recreational activities adopted during college years such as binge drinking and smoking contribute further to high BP [13]. The stress of pursuing a college education along with the increase in sedentary behaviors during this time period further increase the risk factors for high BP and other cardiovascular conditions [14].

Although behavioral and dietary changes can mitigate future risk, a study of college-aged young adults showed that, although they possessed high knowledge of the risks factors for CVD and high BP as well as a functional understanding of the foods required for a healthy diet and activity levels necessary for cardiovascular health, many perceived themselves to be at low risk despite their lack of compliance to these standards [15]. Further study suggests that, although college students may have knowledge of risk factors, a lack of convenient delivery of intervention information and reinforcement of risk factors may cause college students to underestimate the long-term effects of poor cardiovascular health choices [16].

Mobile health (mHealth) has been identified as an ideal technology to deliver health care interventions to young adults. mHealth refers to the use of mobile devices and respective technologies such as apps as a means to exchange health information in order to encourage positive health behaviors [17]. A pilot study conducted by Poddar et al [18] demonstrated improved self-efficacy in daily calcium intake and self-regulation in college students who were sent educational flyers through email. In another study using Facebook messaging to deliver an evidence-based weight loss program to 52 participants, 97% of the participants who completed a satisfaction questionnaire found the program to be helpful [19]. A larger-scale example that has shown promise is the Choosing Healthy Options in College Environments and Settings (CHOICES) study [20]. This large-scale study demonstrated that an interactive website focused on health promotion had the potential to reduce obesity among young adults over the long term [20].

Developing early interventions to prevent the onset of high BP at earlier ages could significantly reduce both the onset and economic burden of medical costs related to CVD. Findings on the receptivity of health and technology indicate that the use of mHealth apps has the potential to assist with behavioral modification for young adults if the apps are simple and streamlined, minimizing the amount of effort on the part of the individual to access the information [16]. As a result, mobile health care and communication methods may be used to aid in promoting BP reduction interventions for at-risk college-aged individuals and may also be effective at promoting behavioral and dietary changes. Therefore, the purpose of this pilot, feasibility study was to understand the implications and impact of using mobile smartphone delivery messages tailored to the participant’s daily self-measured BP values using the Food and Drug Administration–approved Withings BP machine and app. The long-term goal of this pilot study was to develop tailored interventions to increase awareness of elevated BP in college students in a larger study.

## Methods

### Study Design

This was a single-arm intervention, pilot study to evaluate the feasibility of the mHealth to Optimize BP Improvement (MOBILE) intervention focused on elevated BP. Recruitment was conducted at a large university in the southwest region of the United States. Research flyers were posted around the university campus, and face-to-face recruitment was completed at the student union by 2 research assistants on a weekly basis between March 2019 and September 2019.

An approach script was used to provide a standard set of information to potential participants. Individuals who showed interest were then screened for inclusion and exclusion criteria. The inclusion criteria for this MOBILE feasibility study included being a college student (full-time or part-time) at the recruited university, aged 18 years to 39 years, who had regular access to a mobile smartphone with unlimited text messaging, and with SBP >120 mm Hg and DBP ≥80 mm Hg. Students were excluded from the study if they were currently on antihypertensive medications, pregnant, lactating, or planning to become pregnant during study duration or thought they might be pregnant, or if they reported they had a life-threatening illness or condition.

### Recruitment and Participants

During the recruitment process, if any student expressed interest in participating, the research assistants met with the student; completed the initial screening for the inclusion and exclusion criteria, which involved taking at least 2 BP readings; verified they were not on antihypertensive medication; and confirmed they had access to unlimited text messages on their smartphone. Initial screening was completed in a private area to provide confidentiality to the potential participants at the designated recruitment site. If the potential participant met the inclusion criteria, they were invited to participate in the study, and informed consent was obtained. Those who agreed to participate completed a sociodemographic questionnaire and scheduled a brief education session within the following 2 weeks. The education sessions were limited to 1 to 3 participants to ensure appropriate time was given to each participant. Each educational session took approximately 30 minutes, and during this time, the research assistants delivered a brief educational presentation on high BP and explained what the intervention entailed. Two more BP readings were completed at the start of the educational session, before the presentation. After the presentation was completed, participants were instructed on how to use the BP cuff, created a username and log-in information, and were taught how to share their BP daily with the research team. A return demonstration on the BP cuff usage was required. At the end of the educational demonstration, participants scheduled another meeting with one of the research assistants after 28 days for the last assessment.

### Intervention

The MOBILE intervention consisted of having each participant take their BP using the BP cuff that was provided during the educational session by the research assistants. They were instructed to take at least 2 readings (1 on each arm) at their convenience and to send the BP value to the research assistant once completed. The research assistants would then send preset text messages corresponding to their BP value related to encouraging healthy lifestyle modifications. Table 1 shows an example of the intervention text messages. Each participant was required to take his or her BP daily for 28 days.

**Table 1:** Examples of the tailored text messages for the intervention.

Message type and trigger		Example message
**SMBP^a^ monitoring measurement**		
	SBP^b^ <120 mm Hg or DBP^c^ <80 mm Hg	Your BP^d^ is right on target, within normal range. Keep up the good work!
	SBP 120-129 mm Hg or DBP <80 mm Hg	Your BP is slight highly today, remember to increase your physical activity (30 minutes walking), eat less than 2300 mg of sodium a day, drink less alcohol, and consume more fruits and vegetables
	SBP ≥130 mm Hg or DBP ≥81 mm Hg	Your BP is high today; we strongly encourage you to do the following: increase your physical activity (30 minutes walking), eat less than 2300 mg of sodium a day, and avoid any alcohol consumption.
**Missing report message**		
	Had not received any message from the participant	We have not heard from you today. Do not forget to take your BP this week.

^a^SMBP: self-measured blood pressure.

^b^SBP: systolic blood pressure.

^c^DBP: diastolic blood pressure.

^d^BP: blood pressure.

### Data Collection

Daily BP was collected from each participant, and a text message (intervention) was sent to the participant when his or her BP was received for the day. Participation was measured by receiving 1 BP value from the participant and sending 1 text message a day for 28 days. At the conclusion of the intervention, participants completed a survey regarding the usability and effectiveness of the intervention. The researcher-developed, 8-question survey focused on determining the effect of the intervention on BP awareness; reported lifestyle changes (2 questions); and usability issues such as frequency, content, and helpfulness of text messages (4 questions). In addition, participants were asked to provide feedback on any potential changes to the intervention and any other suggestions (2 questions). Survey data were collected within 1 week of completion of the intervention and completed in person at the final assessment visit.

### Data Analysis

Descriptive statistics were used to describe the demographics of the participants and the type of intervention text messages delivered to each participant. A control chart (p chart) was generated to track the participation rate by day. A mixed regression model was used to evaluate the changes in participants’ BP over the study period, with covariates of age, BMI, and gender. Analyses were performed using SPSS version 24 (IBM Corp). Data are reported as mean (SD), with a P<.05 considered significant.

The postintervention survey data were analyzed based on participants’ responses to the specific questions using principles of quantitative content analysis. The research team read and reread the survey responses in order to quantify and analyze the presence of recurring words and phrases. As each survey was analyzed, specific words and phrases were coded, and subsequent surveys were reviewed for the presence of these codes. In addition, as new words and phrases were discovered in the survey answers, they were identified as additional codes. These were then examined for patterns and frequency across the questions and subsequently clustered as code categories. Once the survey answer text was clustered into categories, the research team was able to focus on and code for the specific words or patterns that addressed the questions. For example, the analysis revealed themes such as “awareness of BP,” “risk reduction,” and “changes in diet.” The results were then delineated by each of the 8 survey questions.

### Ethical Considerations

The study received exempt approval from the University of Nevada Las Vegas Institutional Review Board (IRB number 1207565) where all recruitment and study procedures were conducted. Written informed consent was obtained from all participants prior to initiation of data collection. Research assistants explained the informed consent in detail and provided opportunities for the potential participants to ask questions and receive clarification. All study data were deidentified, and each participant was given an anonymous numeric identifier. Data were maintained on a password-protected computer in an encrypted file, and only the research team members who were directly involved in the study had access to the files. The participants received a total of US $50 as compensation for their participation.

## Results

### Participant Characteristics

A total of 11 participants were recruited at baseline, and 2 participants were excluded from the analysis because 1 of them withdrew after day one and another did not participate in the educational session. Among the 9 participants, 56% (5/9) were Asian American, followed by 22% (2/9) who were African American, 11% (1/9) who were Hispanic, and 11% (1/9) who were mixed race. The majority were male (6/9, 67%) and used an iPhone smartphone device (7/9, 78%). The participants’ ages ranged from 18 years to 33 years, with a mean age of 22.64 (SD 4.54) years, and the majority (5/9, 56%) were between 21 years and 29 years old (Table 2). Among the 9 participants, 56% (5/9) were overweight (BMI=25-29.9 kg/m^2^), and 1 (11%) was obese.

**Table 2:** Demographics, type of phone used during the intervention, and baseline BMI of the 9 participating college students in Las Vegas, NV who had elevated blood pressure over 28 days.

Characteristics			Results
Age (years), mean (SD)			22.64 (4.54)
**Age (years), n (%)**			
	≤20	2 (22)	
	21-29	5 (56)	
	≥30	2 (22)	
**Race/ethnicity, n (%)**			
	Asian	5 (56)	
	African American	2 (22)	
	Hispanic	1 (11)	
	Mixed	1 (11)	
Gender (male), n (%)			6 (67)
**Phone type, n (%)**			
	iPhone	7 (78)	
	Android	2 (22)	
**BMI (kg/m^2^), n (%)**			
	<25	3 (33)	
	25-30	5 (56)	
	>30	1 (11)	

### Engagement and Participation Rate

**Figure 1:**
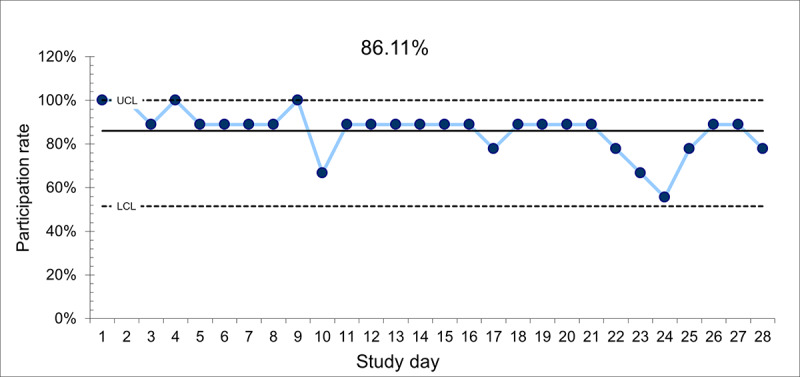
The average daily participation rate over 28 days (N=9) of college students with elevated blood pressure in Las Vegas, NV. LCL: lower confidence limit; UCL: upper confidence limit.

The average daily participation rate was 86.1% (Figure 1). “Participation” was operationalized as the percentage of students who submitted their BP reading to the mobile app on a given day, which varied over the course of the study. For example, during the 28-day study period, the daily participation rate (percent of study participants who reported their BP) ranged from 5 (56%) to 9 (100%) of the 9 participants. The participation rate approached 90% on 17 of the 28 days, as 8 of the 9 study participants (89%) reported their BP. Additionally, we had 100% (9/9) participation on 4 of the 28 days. A total of 218 text messages were sent over the 28-day course of the study. “Your BP is right on target, within normal range. Keep up the good work!” was the leading text message sent (n=40), followed by “Your BP is within the normal range. You are doing amazing.” (n=32). However, 2 of the participants received 16 and 10, respectively, of these messages.

**Figure 2:**
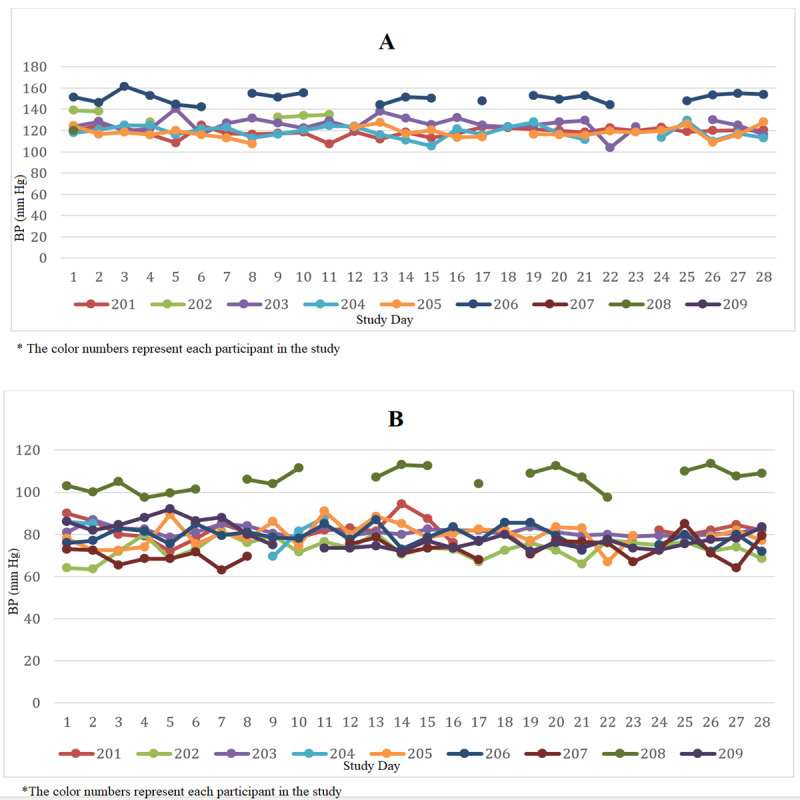
The (A) systolic blood pressure (BP) and (B) diastolic BP level for each participant during the 28-day study period in Las Vegas, NV.

We measured BP at least twice before the start of the intervention, and the participants were tasked with measuring their BP daily for 28 days. Figure 2 depicts the BP for each participant during the study period. Based on the mixed regression test outputs, there were no significant changes in BP over the 28 days. However, we did find that male participants with higher BMIs had significantly higher SBP and DBP.

### Participant Perspectives on the Intervention

Of the 9 participants, 8 completed 100% of the survey’s questions that focused on their perspective of the usability and effectiveness of the MOBILE intervention (Table 3).

All participants (n=8) responded affirmatively that the intervention was successful at increasing their awareness of their individual BP levels and found the text messages to be helpful. When asked for more detail regarding why they found the messages helpful, 4 comments aligned with the first question and indicated the texts helped remind them to measure their BP, understand the category of their BP, and understand the potential outcomes:

Yes they gave me guidance to assist with decreasing and maintaining a more favorable blood pressure.

P201

In addition, 50% (4/8) specifically mentioned they found the messages related to risk reduction, such as diet and exercise and physical activity, to be helpful:

Yes, help to remind me to be conscious of what I eat and to be physically active.

P209

**Table 3:** Feasibility questionnaire after the participants completed the intervention at 28 days (n=8).

Question and response options				Results, n
**1. Did the intervention increases your awareness of your blood pressure levels?**				
	Yes			8
**2. Did you find the text messages helpful?**				
	Yes			8
	**Why or why not?**			
		Awareness	4	
		Risk reduction	4	
		Negative	2	
**3. Do you think the frequency of the text messages being sent was appropriate?**				
	Yes			8
	**If not, how often would you prefer to receive the text messages?**			
		Positive	2	
		Inconsistent	2	
		Wanted more	1	
		Constant feedback desire	1	
**4. What are your thoughts regarding the length of the intervention (4 weeks)? Too short, too long, just right**				
	Just right			6
	Preferred longer			2
**5. Have you made any changes to your lifestyle?**				
	Yes			8
	**If yes, what?**			
		Diet and exercise	3	
		Changes in exercise	3	
		Changes in diet	2	
**6. What would you like to see differently with the intervention to increase your engagement and participation?**				
	More resources			7
	Exercise plan			2
	Nutrition suggestions			4
	More personalized			3
	No suggestions			1
**7. What was the ease of use regarding the blood pressure machine? Any technical difficulty?**				
	Accessibility			1
	Portability			1
	Ease of use			5
	Not compact			1
	Connectivity problems			2
	Failure to work			2
**8. Anything you would like to share with the researchers regarding your participation.**				
	Good experience			3
	Share more data/analysis			2
	More variety in messages			1

All participants (n=8) reported making lifestyle changes during their participation in the intervention: 3 indicated they changed their exercise patterns, 2 made improvements in their diet, and 3 made positive changes to both their diet and exercise:

I changed my diet and amount of exercise by working out at least four times a week.

P201

I actually started exercising more towards the end of the study after seeing my B/P was constantly elevated.

P205

A number of questions focused on the ease of use of the intervention. All (n=8) participants indicated they found the frequency of the text messages to be appropriate:

The daily texts were enough.

P201

Two participants wanted more text messages that included more detail, such as meal plans and more constant individual feedback:

Some more resources would be nice, such as meal suggestions or other educational resources.

P202

Regarding the length of the intervention, 6 of the 8 participants indicated the 4-week span of the intervention was just right. However, 2 participants preferred a longer intervention and felt 4 weeks was not quite enough time.

I think the length of the intervention was just right because you could see the improvements and changes.

P206

I think three months would have been better...longer length in time for the study would have helped see either a change in B/P or stagnation.

P207

Feedback regarding the ease of use of the BP machine was mixed. Participants (n=5) had positive experiences, as they reported it was easy to use and did not encounter any technical difficulties:

BP cuff was easy to use...user friendly.

P204

However, others indicated they had connectivity problems and that the cuff did not work. One participant reported the cuff was not compact enough.

50% of the time it worked/failed. Sometimes it would not connect...

P207

Participants were also asked what they would like to change or add to the intervention to increase their engagement and participation. The responses indicated they wanted more guidance regarding lifestyle changes such as exercise plans (n=2) and diet or meal suggestions (n=4).

I would like to see examples of exercises and the time (durations) of the workout...

P201

Some more resources would be nice, such as meal suggestions or other educational resources.

P203

In addition, they indicated more personalized and individualized information would be helpful (n=3):

Receiving more personalized messages because they seemed premade.

P207

Weekly or biweekly progress notes would seem appropriate and may even motivate me to keep going.

P205

Finally, the students indicated they found participating in the study to be a positive experience:

I found this research study participation to be a good experience and a great teaching tool for health promotion.

P204

They provided additional suggestions such as using an online database for results and providing variation in the text messages.

## Discussion

### Principal Findings

Based on our findings, the mHealth-supported MOBILE intervention for BP monitoring and tailored text messaging was feasible to implement, as our study indicated high rates of engagement among the college student sample. During the study time frame, 9 participants used MOBILE to obtain and report their BP over the course of the 28-day intervention from 56% to 100% of the time, averaging an 86% participation rate. Our feasibility study found a high daily participation rate, which is promising given the 4 weeks required for daily data collection. Among the 28 days, the majority of the participants reported their BP on 17 of 28 days (89%), and there was 100% compliance of BP reporting 4 of 28 days. This can be interpreted as, on 21 of 28 days, the participation rate was >90%. This is high for a daily intervention. These results also reflected the participants’ perspectives regarding the intervention. Specifically, 6 of the 8 participants stated that the 4-week intervention was sufficient, yet surprisingly, some of the participants actually preferred a longer intervention. Understanding the participants’ perspectives related to the length of time for the intervention is useful and can help inform future testing of the intervention with a larger randomized controlled trial to determine effectiveness and efficacy in this population with elevated BP.

The study results also supported the importance of an individualized, tailored approach. Although the intervention was successful at increasing participants’ awareness of their individual BP levels and all found the text messages to be helpful, the majority of the participants reported wanting more resources to make the lifestyle changes to reduce their high BP as well as variation and individualization of the messaging. Participants recommended providing more guidance regarding lifestyle changes related to nutrition and physical activity. They suggested adding exercise plans and meal suggestions that would be delivered through text messaging. Their feedback is informative for future implementation of a large randomized controlled trial and emphasizes the importance of an initial assessment regarding the extent of information appropriate to each participant based on their needs (ie, nutrition, physical activity, knowledge). This implementation will keep some of the standardized baseline intervention but also address individualization that would benefit the participants during and after the intervention.

Our study aligns with other studies involving mHealth interventions resulting in similar levels of high engagement and acceptability. A recent study by Quintiliani and colleagues [21] found an mHealth intervention feasible and useful for weight management and counseling among breast cancer survivors. Similarly, mHealth approaches were used successfully to address or prevent excessive drinking in college students [22]. Likewise, Kaplan et al [23] recognized that mHealth enables individuals to achieve a significant BP reduction through in-depth interactions that encouraged behavioral changes and participant engagement with treating their condition. Similarly, Cianflone et al [24] demonstrated that their participants were able to achieve reduction in both SBP and DBP, by 4.7 (SD 1.2) mm Hg and 3.1 (SD 0.8) mm Hg on average, respectively, using an mHealth-delivered lifestyle change program. In contrast, a systematic review conducted by Jamshidnezhad and colleagues [25] indicated that existing research regarding mHealth for hypertension intervention lacks consistency in its impact on self-care behavior of patients with hypertension. This review suggests the need for further investigation into the potential issues in the study designs and interventions and a focus on rigorous intervention implementation.

Although there was no significant reduction in BP over the 28 days, all participants who responded to the postintervention questionnaire (n=8) reported making positive lifestyle changes in diet or exercise during their participation in the intervention. Our study did reveal that male participants with higher BMIs had significantly higher SBP and DBP. This is of concern and should be further assessed to intervene appropriately given young men are at higher risk of CVD than young women [26,27]. Potential reasons we did not find a BP reduction include the variations in BP levels resulting from multiple factors such as time of day, food consumption, physical activity, and emotional state. Furthermore, we had a small sample size and only assessed 4 weeks of BP, which, according to the literature, is sufficient to determine if there was a significant reduction but may not be enough to find a relationship [28]. Additionally, despite clear instructions during the educational session on how and when to take their BP, we cannot fully validate if there were adherence issues since the participants were self-monitored.

Despite our small sample size, we were able to recruit racially and ethnically diverse participants. A significant portion of our study sample reflects segments of the general population who experience health disparities, particularly related to conditions such as CVD and hypertension, and decreased access to care. Further efforts to include a diverse sample are likely to be successful given the overall diversity of the campus used for recruitment for this pilot study.

### Implications for Practice

This pilot study lays the groundwork of mHealth as an effective medium to increase awareness and guide participants to modify their lifestyles to improve BP in college-aged adults. High BP requires lifetime management with lifestyle modifications, and monitoring BP daily (as in this intervention) enables individuals to understand their daily BP levels and trends and may encourage behavioral changes. Health care professionals play a fundamental role in educating individuals and providing optimal feedback for adjustment when necessary. The MOBILE intervention with mHealth can be used as a primary lifestyle modification tool in both outpatient and inpatient settings for those who have elevated BP and are initially diagnosed with hypertension, providing the feedback they need to build a strong heart-healthy foundation.

### Limitations

This study was not without limitations. The study involved a small sample size and lacked a control group; however, given this is a feasibility study, this was appropriate. There were internet connectivity issues reported by some of the participants who used Android phones; however, one aspect of this feasibility study was to determine the technical issues that participants may encounter to help us anticipate and improve in a larger randomized controlled trial. Additionally, all participants were required to have a smartphone device with unlimited text messaging to participate, which may have led to bias in our sample; however, given this is an mHealth intervention study, this was an appropriate inclusion criterion due to our limited resources. All attempts will be made to avoid bias related to resources that may expand our recruitment and target population in future studies. It is worth noting that we acknowledge this as a potential bias; however, this was a nonissue during recruitment because our target population, namely college students, all had a smartphone device with unlimited text messages.

### Conclusions

This pilot study provides support for the feasibility of an intervention with high participation rates and appropriate approaches regarding the length of time and frequency of text messages. Based on the suggestions and feedback from participants, improvements and alterations will be made to the mHealth BP reduction intervention that can inform successful implementation when a future randomized controlled trial is conducted with a larger sample size. As a whole, this was a positive experience for the participants that made them aware of their BP and the potential steps to reduce their BP and lifestyle in its entirety.

## Abbreviations

BP: blood pressure
CHOICES: Choosing Healthy Options in College Environments and Settings
CVD: cardiovascular disease
DBP: diastolic blood pressure
MOBILE: mHealth to Optimize BP Improvement
SBP: systolic blood pressure
